# Targeting Ligand Specificity Linked to Tumor Tissue Topological Heterogeneity via Single-Cell Micro-Pharmacological Modeling

**DOI:** 10.1038/s41598-018-21883-z

**Published:** 2018-02-26

**Authors:** Aleksandra Karolak, Veronica C. Estrella, Amanda S. Huynh, Tingan Chen, Josef Vagner, David L. Morse, Katarzyna A. Rejniak

**Affiliations:** 1 Integrated Mathematical Oncology, H. Lee Moffitt Cancer Center & Research Institute, Tampa, FL USA; 2 Cancer Physiology, H. Lee Moffitt Cancer Center & Research Institute, Tampa, FL USA; 3 Analytic Microscopy Core, H. Lee Moffitt Cancer Center & Research Institute, Tampa, FL USA; 4 Bio5 Institute, The University of Arizona, Tucson, AZ USA; 5 Department of Oncologic Sciences, University of South Florida, Tampa, FL USA

## Abstract

Targeted therapy has held promise to be a successful anticancer treatment due to its specificity towards tumor cells that express the target receptors. However, not all targeting drugs used in the clinic are equally effective in tumor eradication. To examine which biochemical and biophysical properties of targeted agents are pivotal for their effective distribution inside the tumor and their efficient cellular uptake, we combine mathematical micro-pharmacological modeling with *in vivo* imaging of targeted human xenograft tumors in SCID mice. The mathematical model calibrated to experimental data was used to explore properties of the targeting ligand (diffusion and affinity) and ligand release schemes (rates and concentrations) with a goal to identify the properties of cells and ligands that enable high receptor saturation. By accounting for heterogeneities typical of *in vivo* tumors, our model was able to identify cell- and tissue-level barriers to efficient drug uptake. This work provides a base for utilizing experimentally measurable properties of a ligand-targeted agent and patient-specific attributes of the tumor tissue to support the development of novel targeted imaging agents and for improvement in their delivery to individual tumor cells.

## Introduction

Recent advances in identification of tumor specific biomarkers allowed for expansion of targeted therapies that act on particular molecular targets present in the tumor cells, but absent or expressed at lower levels in normal cells. Since these chemical compounds show lower potency against normal cells than tumor cells, the systemic drug-related toxicity is greatly reduced. Several targeting drugs have been approved for clinical use^1^. However, tumor recurrence and drug resistance have still been observed in some patients that were selected for the targeted therapeutic treatments based on their molecular matching^2,3^. Thus the necessity to develop more effective targeting treatments continues.

Clinical success or failure of targeted therapy depends heavily on whether the drug molecules are able to reach all tumor cells (the process of pharmacokinetics, PK) and engage with their molecular targets to invoke the desired therapeutic effect (the process of pharmacodynamics, PD). Conventional PK/PD analyses assess treatment efficacy on the organ or tissue level. The actual processes that take place at the level of a single cell or a single receptor are difficult to measure or visualize *in vivo* in real time. Therefore, there is only a limited mechanistic understanding of how drugs behave *in vivo* which is a major impediment to developing more efficient anticancer treatments and more effective treatment administration schemes^4^.

The insufficient penetration of drugs is especially important in oncology, since tumors are known for being highly heterogeneous on multiple levels^3^. Morphological and cytological variations between different sections of a tumor are well recognized and routinely used by pathologists for tumor grading. Tumor clonal development resulting in genetic alterations inherited or ascending during tumor growth has also been identified as a cause of cellular diversity within the tumor^5^. In addition, a highly disorganized tissue architecture comprising of regions of densely packed cells and rich stromal components, together with non-optimal tumor vasculature leads to steep gradients in targeted drug concentrations and may generate regions that are unexposed to the drug^6–8^. The complexity of tumor microenvironment has also been associated with the emergence of drug resistance^7,9^. Such multiple levels of tumor heterogeneity make it hard to dissect which aspects are in fact pivotal for the intratumoral distribution process for a given targeted drug^2,10^. Thus, the intratumoral heterogeneity remains a great obstacle to effective penetration of targeted drugs or targeted imaging conjugates^11–13^.

The impact of tumor heterogeneity on the process of drug delivery to individual cells is challenging to study *in vivo*^11,14^. However, recent advancements in molecular imaging techniques made experimental tracking of cellular uptake of imaging and drug molecules attainable^15–18^, and gave rise to the emerging field of *in vivo* single-cell pharmacology^17,19–22^. Classical PK/PD mathematical modeling treats the tumor tissue as a homogenous compartment and neglects any tumor heterogeneities. Although, constant improvement in intravital imaging methods provided experimental data at a single cell level that motivated the development of a number of new mathematical models addressing variability in PK/PD processes at a cell-to-tissue scale^16,23–29^.

However, one of the less-studied aspects of tumor heterogeneity is the variability in tumor tissue cellular architecture and the non-uniform expression of target receptors, both having a strong influence on efficacy of targeted therapies. To account for that, we deliberately chose to use digitized intravital fluorescence images of a mouse xenograft tumor to inform our model. This allowed for calibration of the previously developed *microPKPD* (microscale PK/PD) model^30–32^ to a particular tumor and a particular imaging ligand. Using this calibrated model as a baseline, we compared the uptake efficacy of the hypothetical targeted molecules by altering their diffusivity, binding affinity, intravascular concentrations and extravasation rates. Our ultimate goal was to characterize the role of tumor tissue heterogeneity on ligand uptake on a microscopic single-cell level. The model determined which modifications of physicochemical properties, dosage and extravasation rates of a ligand molecule would provide optimal cellular uptake for a given tumor tissue topology and receptor expression.

## Materials and Methods

### Mathematical model

**Figure 1:**
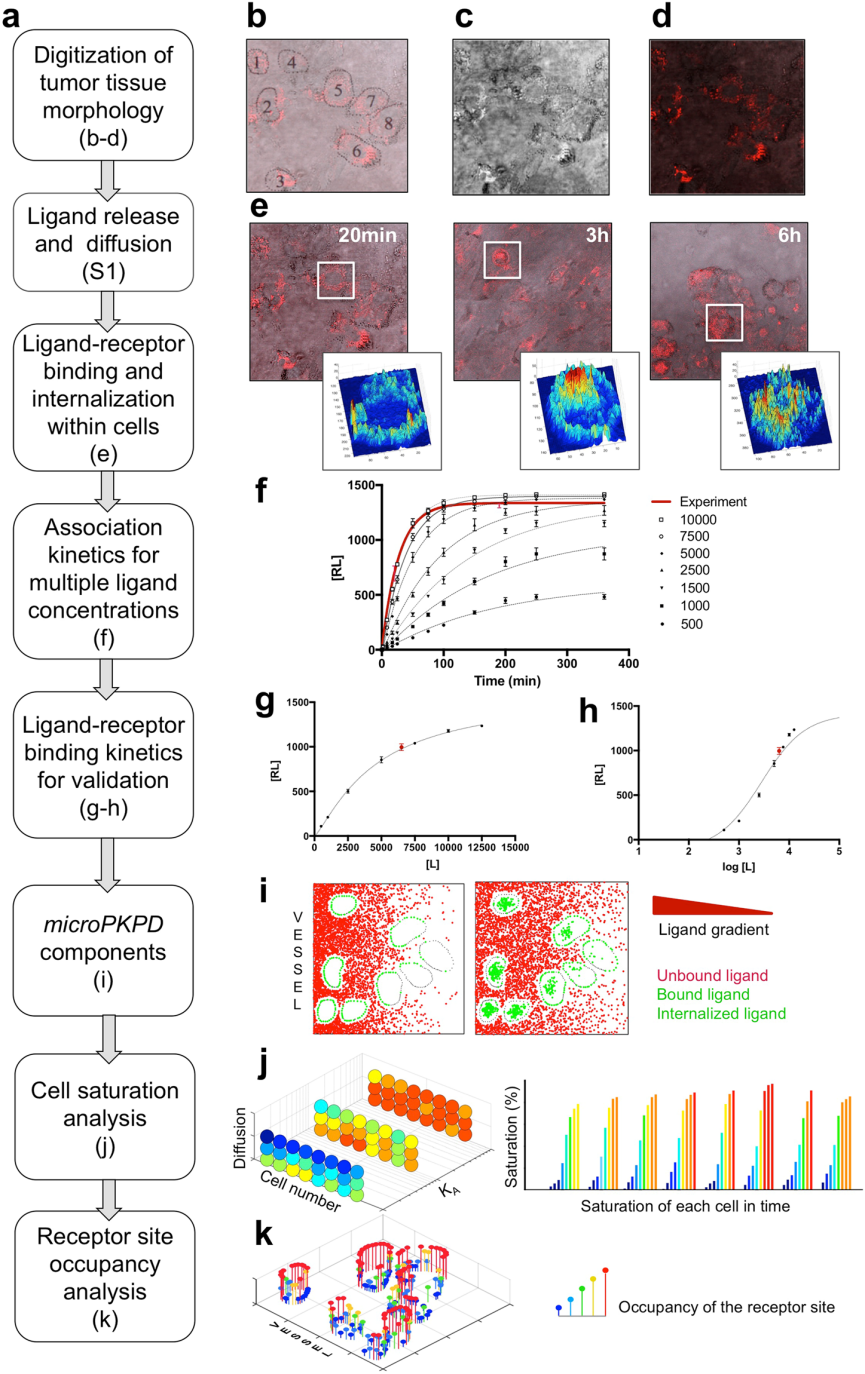
Model calibration. (**a**) A flowchart summarizing *microPKPD* calibration with experimental data and methods for model validation and analysis. (b-d) Digitized tissue morphology: (**b**) identification of individual cell shapes and active receptor expression based on co-registration of (**c)** a bright field image and (**d**) a far-red fluorescence image of the experimental tissue sample; (**e**) spatiotemporal quantification of targeted ligand binding and uptake on the single-cell level at 20, 180 and 360 minutes; insets show ligand intensity heat maps for indicated cells; (**f**) simulated association kinetics fitted to the experimental data; (**g**,**h**) simulated binding kinetics in linear and logarithmic scales, respectively; (**i**) *microPKPD* model components; (**j**) visualization of receptor saturation on a cell level; (**k**) visualization of individual receptor saturation. All simulations were repeated (n = 3) three times, and the average values are shown. The bars in panels (**f**–**h**) represent s.d. values.

Our *microPKPD* modeling framework comprises the discrete representation of imaging agent molecules that interpenetrate the explicitly defined tumor tissue topology, and interact with individual tumor cells via their distinct membrane pseudo-receptors utilizing the quantitative binding kinetics. The *microPKPD* model components, a calibration scheme and the analysis methods are presented in Fig. 1a.

#### Virtual tumor tissue

The model uses tissue morphology digitized from a two-dimensional (2D) intravital fluorescence microscopy image of a human pancreatic xenograft tumor in a dorsal window chamber on a SCID mouse (Fig. 1bc). The explicitly defined virtual tumor tissue is comprised of cells {*C*^*l*^}_*l* = *1*…*N*_ (*l* is a cell index) that are non-motile and non-proliferative due to the short, 6 hours, simulation time frame. The tissue boundary conditions allow for a time-dependent influx of ligands along the left boundary (a capillary), non-leaking top and bottom boundaries, and outflow along the right boundary (tissue clearance). All model parameters are listed in Table 1 and Supplement S1.

**Table 1:** Summary of the *microPKPD* model parameters.

Parameters	Measured experimentally: Cy5-TLR2L ligand and SU.86.86 cells	Fitted to experimental data: Cy5-TLR2L ligand and SU.86.86 cells
Model calibration	(1) Binding affinity between TLR2 receptors on SU.86.86 cells and Cy5-TLR2L [Supplement S3] (2) Number of TLR2 receptors in 3D: 19,300 [Supplement S2]	(1) Diffusion of Cy5-TLR2L, D = 2.5 × 10^−5^ mm^2^/s [Supplement S4] (2) Number of TLR2 pseudo-receptors in 2D: 150–215 [Supplement S2]
	Varied in simulations to explore ligand properties	
Simulation study	(1) Affinity: K_A_ = 1% or 10% or 100% (2) Release time: fast = 1 minute or slow = 3 hours	(1) Diffusion: D = 2.5 × 10^−6^ mm^2^/s, 2.5 × 10^−5^ mm^2^/s, or 2.5 × 10^−4^ mm^2^/s (2) Concentration: [L] = 500, 1000, 1500, 2500, 5000, 7500, or 10000

Listed are model parameters that were either measured experimentally, fitted to experimental data or broadly varied in the model simulation studies.

#### Cell pseudo-receptors

Each individual 2D cell C^*l*^ is identified by membrane pseudo-receptors *C*^*l*^ = {(*X*_*i*_*, Y*_*i*_)^*l*^*, A*_*i*_^*l*^*, B*_*i*_^*l*^}_*i *= *1*…*M*_^*l*^, with coordinates (*X*_*i*_,*Y*_*i*_)^*l*^, receptor affinity *A*_*i*_^*l*^ and its saturation level *B*_*i*_^*l*^. The number of pseudo-receptors, *M*^*l*^, depends on cell size identified from experimental images (Fig. 1d). The receptor saturation level is determined from the coarse-graining of the cell membrane circumference. Based on the total number of membrane receptors per cell and receptor size, we estimated that five pseudo-receptors, each represented by one boundary point, hold up to five pseudo-ligands each (Supplement S2).

#### Discrete ligand molecules

The concentrations of fluorescently labeled ligands are represented by numbers of discrete molecules [*L*] which vary between 500 and 10,000. These amounts were selected to fit *in vivo* data (Fig. 1f). The molecules are supplied from a capillary via a fast or slow release scheme. Interstitial transport of the ligand is modeled as Brownian motion with an effective diffusion coefficient *D* between 10^−4^ and 10^−6^ mm^2^/s. This is consistent with experiments and literature^24^, and takes into account agent biophysical properties, such as size and solubility^33^.

#### Binding affinity

Binding affinity is defined as the probability at which the ligand binds to the receptor after successful recognition. We consider three settings: strong probability of binding (100%), moderate (10%), or weak (1%), resulting in three values of the pseudo-association constant (*K*_*A*_): 100, 10, and 1, respectively.

#### Binding process

The binding condition (BC) between the agent and receptor requires that: (i) the ligand molecule is in close proximity to the receptor, *i.e*. ||(*x*_*j*_*, y*_*j*_)_*n*_ − (*X*_*i*_*, Y*_*i*_)^*l*^|| < *r*_*min*_, where (*x*_*j*_*, y*_*j*_)_*n*_ are the coordinates of the *j*^*th*^ ligand at *n*^*th*^ simulation step, (*X*_*i*_*, Y*_*i*_)^*l*^ are the coordinates of the *i*^*th*^ receptor of the *l*^*th*^ cell, and *r*_*min*_ is the minimum distance; (ii) the receptor is not saturated, that is the number of ligands already bound to this receptor is below *B*_*i*_^*l*^; and (iii) the probability of binding meets the affinity criterion *A*_*i*_^*l*^. This procedure mimics the distance-dependent electrostatic and hydrophobic interactions initiating the recognition and binding processes *in vivo*. Binding to one receptor does not alter the affinity or recognition process of sites on adjacent receptors.

The motion of a ligand molecule *(x, y)* at the *n* + 1 time point is defined by Equation 1:

1 $${(x,y)}_{n+1}=\{\begin{array}{ll}{({X}_{i},{Y}_{i})}^{l} & (A)\,if\,BC\,is\,satisfied\\ {(x,y)}_{n}+\sqrt{2D{\rm{\Delta }}t}{\omega }_{n+1} & (B)\,if\,the\,molecule\,is\,in\,the\,interstitial\,space\\ {(x,y)}_{n} & (C)\,if\,the\,molecule\,crosses\,cell\,boundary\,randomly,\end{array}$$

where Δ*t* is a time step, ω is a randomly chosen direction of motion, and *D* is the effective diffusion coefficient. Note, that cell membranes are non-penetrable for the ligands unless the ligand is successfully recognized by a receptor (Equation 1A). Therefore, if due to random motion (Equation 1B) a ligand molecule crosses the cell boundary without satisfying BC, that molecule’s position will remain unmodified (Equation 1C).

#### Receptor-mediated endocytosis

The process of receptor-mediated endocytosis is modeled as internalization of a receptor-ligand complex into the cell in a time-dependent manner, using a minimum residency time of 20 min on the cell membrane, as estimated from experimental data (Fig. 1e). The internalized ligands are randomly distributed within the cell, mimicking the *in vivo* endocytosis process.

#### Extravasation of ligand into the tissue

Two schemes of ligand extravasation that depend on time of release from vasculature are implemented in the model. For both schemes, the same amount of ligand molecules is released over either 1 minute (fast release) or 3 hours (slow release). Such an approach mimics various routes of administration in anti-cancer therapies.

### Experimental Approach

Our experimental framework employed a dorsal window chamber murine model utilizing the metastatic pancreatic adenocarcinoma (PDAC) cell line SU.86.86 with endogenous expression of TLR2^34^. Intravital fluorescence images of a mouse xenograft tumor were acquired over a time-course following intravenous administration of a fluorescently-labeled ligand-conjugate. We chose to use a high affinity TLR2-specific ligand conjugated to cyanine-5 dye (Cy5-TLR2L). The details of synthesis, purification and characterization of Cy5-TLR2L (Cy5-Mpr-PEGO-Cys(*S*-[2,3-bis(palmityol)oxy-(R)-propyl])-Gly-DSer-PEGO-NH_2_) are given in Supplement S5.

#### Tumor Cell Characterization

The SU.86.86 human pancreatic adenocarcinoma cells were acquired from ATCC in 2007. Prior to beginning the study and after completion, the cells were authenticated and tested for mycoplasma. Cells were authenticated by the Moffitt Cancer Center Molecular Genomics Core using short tandem repeat (STR) DNA typing according to ATCC’s guidelines in^35^. At both times, the cells tested negative for mycoplasma and were determined to be 100% identical to SU.86.86 human pancreatic adenocarcinoma cells (ATCC CRL-1837). During the study, these cells were passaged no more than 7 times from frozen stock. Experimental data confirming TLR2 expression in SU.86.86 cells and determining the *in vitro* receptor number are presented in Supplements S2 and S3.

#### Dorsal Window Chamber Xenograft Tumor Experiments

Immuno-compromised NOD/SCID mice, 22–25 g, were purchased from Charles River Inc. and handled in compliance with the Guide for the Care and Use of Laboratory Animal Resources (1996), National Research Council, and approved by the Institutional Animal Care and Use Committee, University of South Florida, under an approved protocol (Supplement S6). As previously described, the window chamber systems were sterilely implanted on a skin fold of the dorsal side of mice using the tumor droplet method^18,30^. For each mouse, a layer of skin was removed to provide a region of exposed tissue located under the glass cover of the chamber, the glass cover was removed and 1–2.5 × 10^6^/ml SU.86.86 human pancreatic tumor cells suspended in a droplet of 2.5 mg/ml rat tail Type I collagen matrix surrounded by green fluorescent protein (GFP) expressing rat micro vessels (also in collagen) and inoculated onto the region of exposed tissue. The glass cover was replaced and the xenografts were observed twice per week by intravital bright-field microscopy for 10–15 days until tumor cells had proliferated and new vasculature had penetrated into the tumor. Prior to administration of Cy5-TLR2L, 10,000 MW cascade blue dextran (D1976, Thermo Fisher) was administered intravenously and used to visualize tumor vessel patency by microscopy.

#### Intravital Confocal Fluorescence Microscopy

Once the tumor has an established vasculature, 100 nmol/kg of the fluorescent Cy5-TLR2L was injected into the tail vein and monitored for vascular circulation, tumor extravasation and penetration, and tumor cell binding and uptake by intravital confocal fluorescence microscopy. Mice were sedated with 1.5% isoflurane, covered with a warm pad to maintain appropriate body temperature and breathing monitored for the duration of the imaging session. A series of acquisitions were obtained at 20, 180 and 360 minutes post injection. Confocal images were acquired using the Olympus FV1000 MPE multiphoton laser scanning microscope with a 25 × XL Plan N 1.05 N.A. Water immersion objective lens and zoomed in 4×. Spatial distribution of Cy5-TLR2L in the tumor and adjacent normal tissue was obtained by exciting the dye with a Red HeNe2 Laser at 633 nm excitation wavelength and monitoring the emission spectra with a Barrier filters BA655-755. Bright field images were acquired using the transmitted detector1 channel. The confocal images were converted to tiff format using FV10-ASW 3.1 Viewer.

#### Ligand association and binding kinetics for linking simulated results with experimental data

The receptor-ligand binding as a function of time is quantified by fitting the association kinetics in Equation 2 to simulated data that represent the averaged saturation per tissue area:

2 $$B={B}_{0}+({B}_{max}-{B}_{0})/(1-{e}^{-kt}),$$

here, *B* corresponds to a receptor saturation parameter, an analogue of ligand-receptor complex formation [*RL*], with values between initial saturation *B*_0_ and the maximum saturation *B*_*max*_; *k* is a reaction rate constant; *t* is time. All fittings were performed with GraphPad Prism^36^. The linear and logarithmic specific binding kinetics are given by Equations 3 and 4:

3 $$B=({B}_{\max }{[L]}^{h}/({K}_{D}^{h}+{[L]}^{h}),$$

4 $$B={B}_{0}+({B}_{\max }-{B}_{0})/(1+{10}^{([L]-Log{K}_{D})}),$$

where *B*, *B*_*0*_ and *B*_*max*_ are saturation parameters, *K*_*D*_ is a dissociation constant, *h* is the Hill slope defining the steepness of the fitting curve and [*L*] is ligand concentration.

### Data availability statement

The mathematical model code and the testing data are available from the authors upon request or from the following website: http://labpages.moffitt.org/rejniakk/LabTools.html.

## Results

To comprehensively understand the pharmacokinetics of tumor targeted agents on the cell-to-tissue level, we extended our *microPKPD* mathematical model^30,31^ by including the quantitative binding kinetics between tumor cell membrane receptors and ligand molecules, and by incorporating into the model the explicitly defined tissue topology. This extended model was first calibrated with experimental data, and then used to investigate which intrinsic (molecule-based) and extrinsic (tissue-based) conditions lead to efficient ligand binding and internalization.

### Model calibration to pancreatic tumor cells and Cy5-TLR2L

Our experimental framework employed a dorsal window chamber murine model. The metastatic pancreatic adenocarcinoma (PDAC) cell line SU.86.86 with endogenous expression of the toll-like receptor 2 (TLR2)^34^ was used together with a high-affinity TLR2-specific ligand conjugated to cyanine-5 dye (Cy5-TLR2L). The process of model calibration with data from these experiments is summarized in Fig. 1a. A pair of co-registered images of a tumor tissue interpenetrated by Cy5-TLR2L was used to define the topology of a computational tissue (Fig. 1b). A bright field image (Fig. 1c) was used to define cell sizes and shapes, and a far-red fluorescence image (Fig. 1d) was used to identify active pseudo-receptors on each cell boundary based on the intensity of the Cy5-TLR2L along the cell circumference.

To match the experimental observations, in which fluorescent ligand was detectable in the vasculature 3 hours after injection (data not shown), ligand extravasation was modeled as a continuous constant-rate influx of ligand molecules during the first 3 hours of the simulated time (slow release). The ligand transport restricted to the interstitial space was represented by Brownian motion (Methods Equation 1) with the estimated diffusion coefficient of 2.5 × 10^−5^ mm^2^/s (Supplement S4).

High affinity binding between TLR2 and Cy5-TLR2L was demonstrated by binding assay (Supplement S3) and reproduced in the model as a 100% probability of successful binding (*K*_*A*_ = 100), provided the binding condition (BC) was fulfilled (Methods Equation 1). Quantification of ligand internalization within cells was determined from far-red fluorescence data acquired at 20, 180 and 360 minutes post administration of Cy5-TLR2L, and presented as heat-surface intensity maps in Fig. 1e. These experimental data allowed us to assess that the average residence time of the entire ligand-receptor complex on the cell membrane before endocytosis lasted about 20 minutes. After internalization, the molecules became randomly placed inside the cell, mimicking the observed *in vivo* intracellular distribution of Cy5-TLR2L in the endosomes.

The association kinetics of receptor binding for a single ligand concentration (Methods Equation 2) were used to fit a time course of images acquired during the first 6 hour of *in vivo* experiments, and to generate the time-dependent association kinetics curve shown as a red line in Fig. 1f. To determine virtual concentrations of the ligand that matched experimental data, a range of virtual ligand concentrations between 500 and 10,000 were applied in simulations, and the obtained data points were fitted to the association kinetics curves (Fig. 1f, black lines). Ligand pseudo-concentrations of 5,000 and 7,500 molecules produced curves that were the closest to the experimentally administered dose (GraphPad Prism, One-phase association kinetics method, correlation coefficients *R*^2^ > 0.98 and *R*^2^ > 0.99, respectively; full statistical analysis is shown in Supplement S7).

In order to ascertain whether the simulated data fit the binding kinetics equations without imposing the values for parameters *B*_*max*_ and *K*_*D*_, the outputs from the association kinetics equation were determined for various ligand concentrations before their plateaus were reached. The fitting results to Equations 3 and 4 are presented in Fig. 1g and h, respectively, showing a very good agreement with the kinetics of binding (GraphPad Prism, Specific binding kinetics method, *R*^2^ > 0.99 for linear and *R*^2^ > 0.97 for a logarithmic scale; full statistical analysis is shown in Supplement S8).

A typical example of the *microPKPD* simulation is shown in Fig. 1i where discrete ligand molecules are supplied from a vasculature located along left domain boundary and form a diffusive gradient, bound to cell membrane receptors and are internalized. Graphical presentation of the process of saturation on the cell level is shown in Fig. 1j, and on the receptor level in Fig. 1k.

### Slow release scheme reveals a non-intuitive relation between diffusivity and affinity

**Figure 2:**
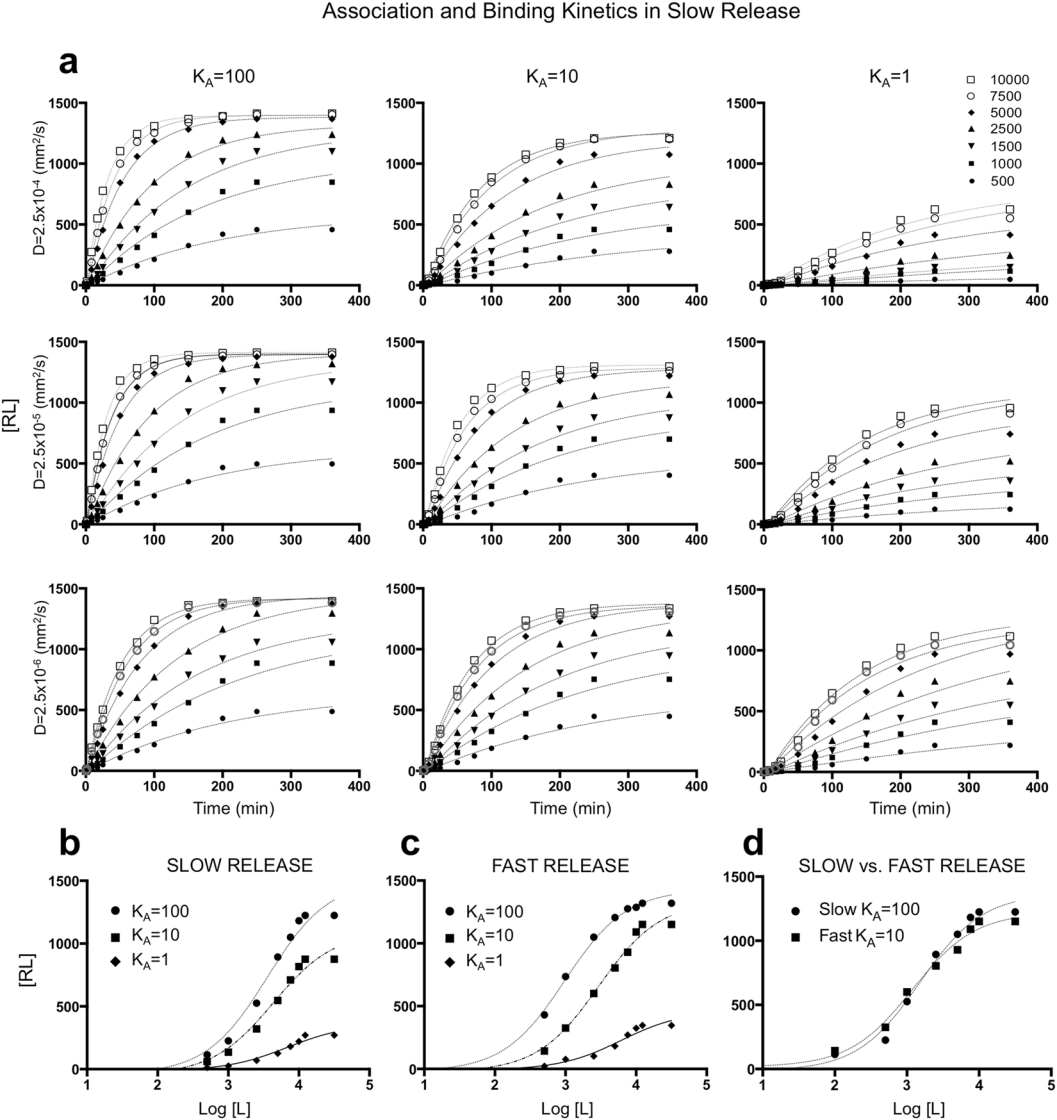
Association and binding kinetics curves. (**a**) Parameter space of the *microPKPD* model for three values of the diffusion coefficient *D*: 2.5 × 10^−6^, 2.5 × 10^−5^, and 2.5 × 10^−4^ mm^2^/s; three values of binding affinity *K*_*A*_: 100, 10, and 1; and ligand concentration values [*L*] between 500 and 10,000. (**b**–**d**) Specific binding kinetics calculated for a diffusion coefficient of *D* = 2.5 × 10^−5^ mm^2^/s, affinities of *K*_*A*_ = 100, 10 and 1, and the slow (**b**) or fast (**c**) release schemes. (**d**) Direct comparison of binding kinetics curves for the slow release scheme with *K*_*A*_ = 100 and the fast release scheme with *K*_*A*_ = 10. For each case (n = 3) three simulations were performed, and the average values are shown. All s.d. values were smaller than 5% and were not shown for clarity of images.

The model calibrated to the properties of Cy5-TLR2L ligand (diffusion coefficient *D* = 2.5 × 10^−5^ mm^2^/s, binding affinity *K*_*A*_ = 100 and slow release scheme) has been subsequently used to determine association kinetics for the broader class of potential imaging agents rising from small rapidly diffusing molecules to larger and less soluble ones. We examined in silico ligand molecules of diffusion values between *D* = 2.5 × 10^−4^ and 2.5 × 10^−6^ mm^2^/s, binding affinity values *K*_*A*_ = 100, 10 and 1, and both slow (Fig. 2a) and fast release schemes (Supplement S9). For each case considered, three independent simulations were carried out and the average value reported. In all cases the standard deviation (s.d.) values were small (below 5%) showing that the model simulation results are robust.

In the case of the slow release scheme, the association kinetics curves for all considered concentrations are characterized by moderate-to-low steepness before their plateaus are reached (Fig. 2a). This is because the availability of the unbound ligand in the interstitial space is limited since only a small number of molecules can extravasate from the vessel at any given time according to the slow release scheme. Nonetheless, in the cases of higher ligand concentrations (above 5,000 molecules) and for high ligand affinity (*K*_*A*_ = 100) the receptors became saturated to their binding capacities for all considered diffusions. For lower concentrations (500 and 1,000) the association kinetics curves reached plateaus below the saturation levels due to inadequate ligand quantity. However, the amount of ligand that has been bound to the receptors reached nearly the maximum feasible level for the amount of ligand that was injected. The concentration of 1,500 ligand molecules, which in theory should saturate the total of 1,430 receptor sites on all cells together (Supplement S2), was in fact not sufficient, even for the agent with high binding affinity. This indicated that the binding efficacy is not directly proportional to the amount of the injected ligand. Some ligand molecules have escaped the domain without being recognized by the receptors due to either not taking the proper path toward the receptors during their random walk, or not spending enough time near the receptors to be recognized. This effect is particularly visible for higher values of *D*.

In general, for all ligands of moderate affinity (*K*_*A*_ = 10) their final levels of receptor saturation decreased with increased ligand diffusion (Fig. 2a, middle column). This suggests that the faster moving ligands left the domain without binding to the receptors. In contrast, the slower ligands remained near the cell receptors long enough to have multiple occasions for binding. The association kinetics curves in these cases are similar to those for high affinity values (Fig. 2a, left and middle column, bottom row). This inverse relation between saturation and diffusivity is the most pronounced in the case of low binding affinity (*K*_*A*_ = 1), although, the final levels of receptor binding did not reach the saturation levels for any of the concentrations considered (Fig. 2a, right column). It is worth noting, that the simulated association kinetics curves agree in their shapes and steepness with those determined experimentally^18,37^. In addition, the affinity-diffusion relationship identified here is supported by experimental observation that smaller ligands require high affinity for efficient binding, whereas slower ligands (for example, larger or less soluble) bind with similar efficacy for a range of affinity values^24,38^.

### Distinct release schemes result in similar binding kinetics for high and moderate ligand affinities

From the same set of simulations, the binding kinetics curves were generated at the specific time points at which receptor occupancy reached 90%, that was chosen following the method described in^39^. This approach allowed us to compare the levels of receptor saturation before they reached plateaus for a fixed diffusion, different values of agent affinity, and both slow and fast release schemes.

The results for the baseline diffusion coefficient of *D* = 2.5 × 10^−5^ mm^2^/s, the three affinity values and slow or fast agent release schemes are shown in Fig. 2b,c. Strikingly, these simulations revealed nearly identical shapes of binding kinetics curves for the moderate affinity-fast release scheme and the high affinity-slow release scheme. These curves are directly compared in Fig. 2d and quantitatively in Supplement S10. Our simulations indicate that similar fractions of receptor sites can be engaged in ligand binding even if the binding affinity is moderate, provided that the ligand molecules are released in a short time (fast release scheme). Therefore, different routes for optimization of biochemical and biophysical properties of the agent can be suggested in order to improve their uptake. For example, if technical or financial conditions prevent modifications in agent structure that would improve binding affinity, one may consider redesigning the method of agent delivery by applying local injections (fast) instead of intravenous (slow) agent release; or by adjusting agent mass since larger molecules will extravasate slower.

### Single-cell analysis of agent uptake reveals a paradox between exposure and binding of fast-diffusing agents

**Figure 3:**
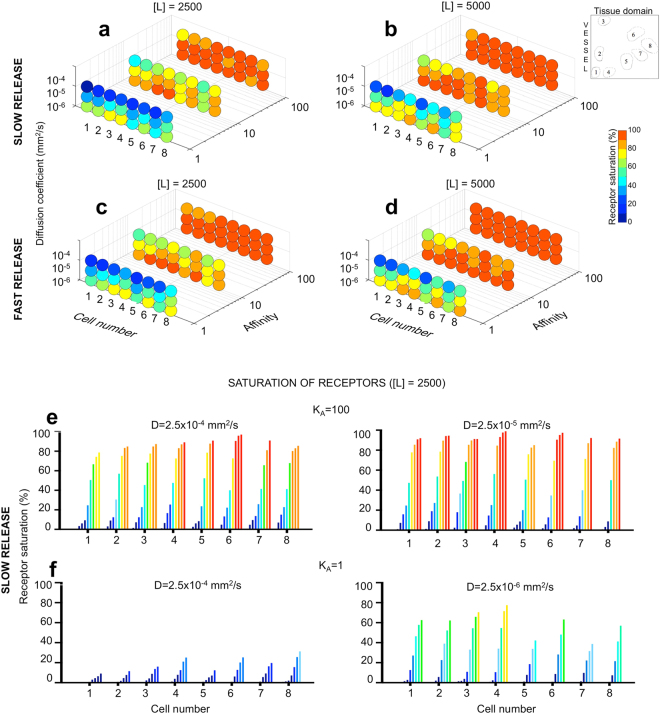
Analysis of receptor saturation on the single-cell level. A cumulative receptor saturation level (percentage) for each individual cell (numbered from 1 to 8) calculated at 6 hours of simulated time (end of the simulation) for all combinations of binding affinity (*K*_*A*_ = 100, 10, 1) and diffusion coefficient (*D* = 2.5 × 10^−6^, 2.5 × 10^−5^, 2.5 × 10^−4^ mm^2^/s) considered in this work: (**a**) Slowly released concentration of [L] = 2,500 molecules; (**b**) slowly released concentration of [L] = 5,000 molecules; (**c**) rapidly released concentration of [L] = 2500 molecules; (**d**) rapidly released concentration of [L] = 5000 molecules. The insets show spatial configuration of cells (1–8) within the tissue and the color bar represents the percentage of saturated receptors. (**e–f**) Receptor saturation level calculated for each individual cell at nine time points; from left to right: 1.5, 10, 20, 30, 45, 90 minutes and 3, 4.5 and 6 hours. In both cases, a slow release of [*L*] = 2,500 ligand molecules was used. (**e**) *K*_*A*_ = 100 and *D* = 2.5 × 10^−4^ mm^2^/s (left) and *D* = 2.5 × 10^−5^ mm^2^/s (right); (**f**) *K*_*A*_ = 1 and *D* = 2.5 × 10^−4^ mm^2^/s (left) and *D* = 2.5 × 10^−6^ mm^2^/s (right). For each case (n = 3) three simulations were performed, and the average values are shown. All s.d. values were smaller than 5% and were not shown for clarity of images.

By representing the ligand as discrete molecules within explicitly defined tissue architecture, both temporal and spatial quantification of agent uptake efficacy on a single cell level can be determined. We traced ligand uptake by each cell separately over the course of 6 hours of the simulated time for all combinations of ligand concentration, diffusivity, binding affinity and release scheme. The representative results for two moderate concentrations (2,500 and 5,000) at the end of simulation are shown in Fig. 3a–d. Since the amount of released ligand molecules for each of these cases exceeded the number of available receptor sites, we expected the majority of receptors to become saturated, at least in the cases of high binding affinity. As predicted, for concentration [L] = 5,000, all cell receptors except one were fully saturated for all diffusion coefficients considered and for both release schemes. This is indicated by columns of red dots in Fig. 3b,d. Interestingly, when a smaller amount of ligand molecules was released and ligand diffusion was fast, the cells that acquired only partial saturation were located the closest to the source of ligand for both release schemes (cells #1–3 in the rear row in Fig. 3a,c). This potential paradox of reduced receptor saturation in cells that were well exposed to the ligand indicates that ligand molecules did not spend enough time near these cells to be recognized by and bound to the membrane receptors. This is illustrated in more detail in Fig. 3e where the progression in cumulative receptor saturation for each of the eight cells is presented as a time series of several time points. It is evident that for slowly released but highly mobile ligand molecules their accumulation has steadily increased in time in all cells, but slower in cells located in closest proximity to the vessel (Fig. 3e, left), while for slower diffusing molecules, receptor saturation in cells within a short distance from the vessel was more pronounced (Fig. 3e, right).

Since the number of injected ligand molecules was sufficient to saturate all receptors, the lack of complete receptor saturation for moderate and low affinity cases shown in Fig. 3a–d was a result of ineffective ligand-receptor binding. However, it is interesting to notice, that the least efficient cases were observed for fast diffusing molecules. This becomes apparent by comparing the receptor saturation levels separately for each column in Fig. 3a–d where top rows correspond to fast diffusion, and bottom rows correspond to slow diffusion of ligand molecules. For example, in the case of ligand concentration of [L] = 2,500 and moderate binding affinity of K_A_ = 10 several cells became fully saturated when agent was diffusing slowly (red cells #3–4, Fig. 3a, bottom of the middle row; and cells: #2–4 and #6, Fig. 3c, bottom of the middle row); while for the fast diffusing ligand, no more than three cells in each case reached 70% of saturation (yellow and orange cells in Fig. 3ac, top of the middle rows). The case of low binding affinity shows even more striking results, and inspection of the time series of the progression of receptor binding shown in Fig. 3f confirms quantitatively that the ligand molecules bind more efficiently if they move slowly enough to be recognized.

### Temporal analysis of individual receptor sites demonstrates a critical role of heterogeneities in tissue architecture

By representing cell receptor sites as discrete entities, the temporal analysis of saturation levels for each individual receptor on every cell within the tissue can be performed. Specifically, we focused on the progression of receptors binding in respect to their locations and with reference to the direction of incoming ligand. We observed that receptors of cells located further from the vasculature can reach higher saturation levels earlier than the cells in close vicinity to the source of injected ligand, which is counterintuitive. This is well visible for moderate affinity and concentration of [L] = 2,500 (middle columns in Fig. 3a,c). In the majority of these cases the saturation level of cell #8 was significantly higher than in the cells located closer to the vessel (cells #1, 2, 5 and 7). This effect was exaggerated for the agent with higher diffusivity.

**Figure 4:**
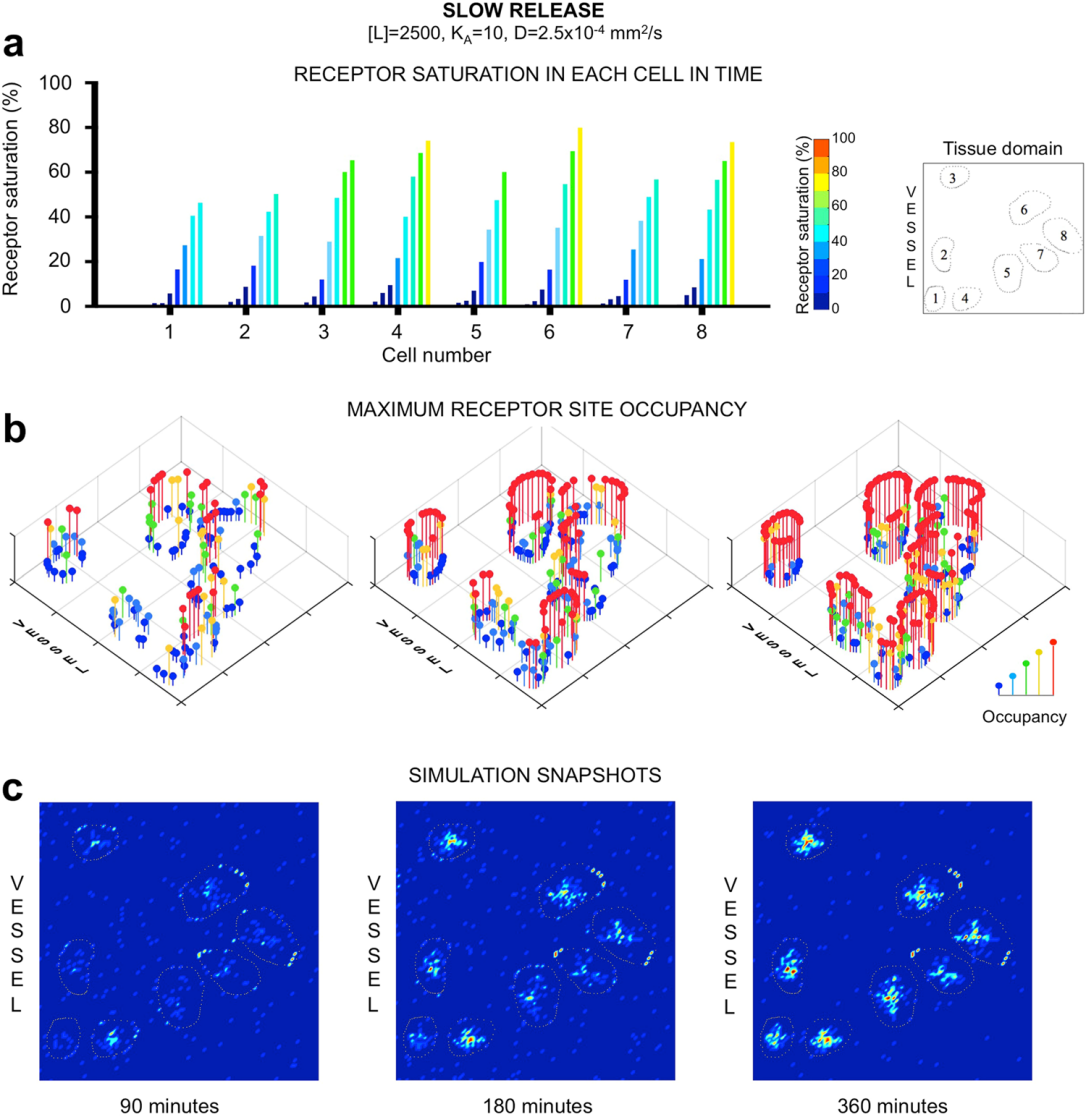
Spatial and temporal analysis of ligand binding and saturation for individual receptors. Considered is a ligand of moderate affinity (*K*_*A*_ = 10) and high diffusion (*D* = 2.5 × 10^−4^ mm^2^/s) released slowly at the moderate concentration ([*L*] = 2,500). (**a**) A time series of cumulative receptor saturation in all eight cells. (**b**) Progression in saturation of individual receptor sites spatially distributed along cell membranes at three different time points: after 90, 180 and 360 minutes of the simulated time. Color-coded pin-like markers indicate how many ligand molecules (between 1 and 5) occupy each receptor. (**c**) Spatial distribution of ligand molecules within the tissue and inside the cells after receptor internalization at the same three time points. Images were rendered to represent fluorescence intensity of ligand concentration. For each case (n = 3) three simulations were performed, and the average values are shown. All s.d. values were smaller than 5% and were not shown for clarity of images.

**Figure 5:**
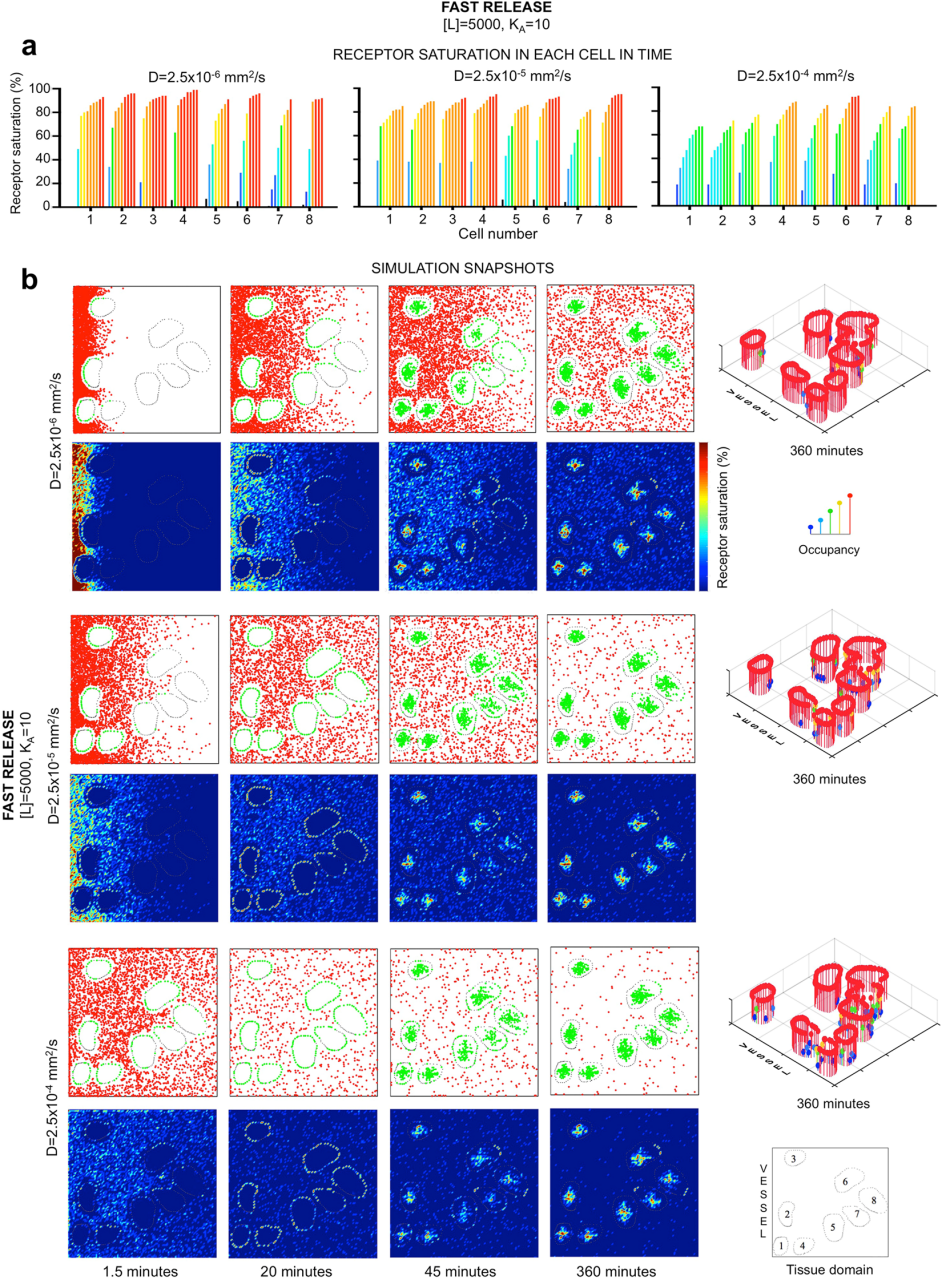
Analysis of ligand molecule movement through the tissue space and its role in receptor saturation. Comparison of three simulations with different diffusion coefficients from *D* = 2.5 × 10^−6^ to 2.5 × 10^−4^ mm^2^/s, binding affinity *K*_*A*_ = 10, concentration [*L*] = 5,000 and fast release scheme. (**a**) A time series of cumulative receptor saturation in individual cells, color-coded to represent a receptor saturation range between 0 and 100%. (**b**) A sequence of snapshots showing spatial penetration of ligand molecules through the tumor tissue; top rows show simulation outputs with red dots representing ligand molecules in the interstitial space and green dots representing bound and internalized ligand-receptor complexes; bottom rows show a fluorescence rendering of the same model outputs to better visualize ligand concentration; the last column shows saturation of individual receptors on each of the eight cells at the end of the 6 hour simulation. For each case (n = 3) three simulations were performed, and the average values are shown. All s.d. values were smaller than 5% and were not shown for clarity of images.

A detailed analysis of one such case (for D = 2.5 × 10^−4^ mm^2^/s, K_A_ = 10, [L] = 2,500 and slow release scheme) is presented in Fig. 4. The time series of receptor binding (Fig. 4a) indicates a steady increase in receptor cumulative saturation for each of the eight cells. However, three spatial snapshots in Fig. 4b taken at 90, 180 and 360 minutes demonstrate high heterogeneity in saturation of individual receptor sites located along cell boundaries. Interestingly, several receptors in cells located further from the vessel have been fully saturated after 90 minutes (Fig. 4b, left panel), which is not the case for cells located close to the vessel. Saturation of receptors in distant cells at this time is a consequence of high diffusivity of the ligand that spreads through the whole domain (compare Fig. 4c, left). Diminished saturation of receptors near the vessel is an effect of moderate binding affinity of the ligand. At the later stages, however, the incomplete saturation of receptors near the vasculature is a result of ligand diffusive movement deeper into the tissue without being recognized by cell receptors. After three hours, the ligand is no longer supplied from the vessel, but can still diffuse through the domain and bind to cell receptors (Fig. 4c, middle row). However, several receptor sites remained unsaturated even after 6 hours (blue and green pins in Fig. 4b, right panel). The similar phenomenon is also observable when the same agent was supplied in higher concentration (Figs 4d and 5b) indicating that partial saturation of certain receptors is a consequence of spatial heterogeneity in cell locations and spatial heterogeneity in expression of cell membrane receptors.

### Spatial analysis of ligand transport corroborates that slow diffusion enhances ligand’s infiltration in limited spaces

The results discussed in the previous sections point out to slow diffusion as more efficient for achieving full saturation of cell membrane receptors. Here, we directly compare three cases that differ only in ligand diffusion coefficient (*D* = 2.5 × 10^−6^, 2.5 × 10^−5^ and 2.5 × 10^−4^ mm^2^/s), but have the same binding affinity (*K*_*A*_ = 10), concentration ([*L*] = 5,000), and are released fast from the vasculature (Fig. 5). Again, slower diffusion resulted in better saturation of all cells (Fig. 5a, left), while faster diffusion prevented full saturation of several cells, including these near the vasculature (Fig. 5a, right).

The snapshots from respective simulations presented in Fig. 5b show spatial and temporal changes in ligand concentration over the 6 hours of simulated time. Quite significant differences in the depth of agent penetration through the tissue are visible as soon as 1.5 minutes post injection. The images with fluorescent rendering of the simulated results show clearly how the gradient of administered agent is forming in each case. The snapshots corresponding to 20 minutes indicate ligand bound to the cell receptors, while the 45 minutes and 6 hours snapshots show subsequent ligand internalization. Final occupancy of each cell receptor is shown in the last column of Fig. 5b. This image indicates clearly that slowly diffusing ligand molecules were bound more efficiently by cell receptors facing the limited space between the neighboring cells (single non-red pins for slow diffusion vs. multiple blue and green pins for fast diffusion). This observation is corroborated by the spatio-temporal analysis of respective snapshots (Fig. 5b). Initially, we observe a drift of saturation within the exposed membranes of cells #5 and #6, since freely diffusing molecules favor the open spaces above cell #6 and under cell #5. However, for cells separated by narrow channels (such as cells #5–8), we see more efficient binding by ligand molecules with a moderate or low diffusion coefficients. In these cases, the ligand molecules may reverse direction and move back and forth resulting in saturation of receptors on these cells. This is not an instance for high mobility ligands, which do not spend enough time in these limited spaces to be recognized by cell receptors. This phenomenon is also observable for ligands of lower affinity; in all cases presented in Fig. 3a–d, the slower diffusing ligands saturate the receptors better than fast moving ligands.

### Comparison of different release schemes shows higher effectiveness of fast released ligands

The amount of ligand that extravasates from the vasculature at any given time (the release scheme) constitutes a pool of molecules within the interstitial space of the tumor tissue that are free to bind to and saturate cell membrane receptors. Here, we investigate what impact the release scheme has on the efficiency and speed of receptor saturation.

**Figure 6:**
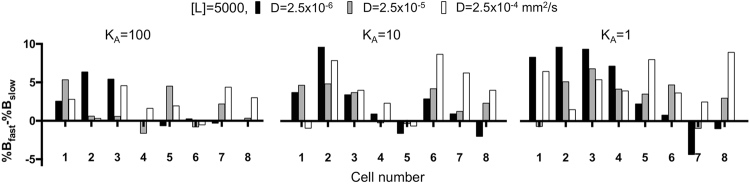
Comparison of single-cell saturation levels for fast and slow release schemes. A difference in percentage of receptor saturation at the single-cell level, calculated as % of *B*_*fast*_ – % of *B*_*slow*_ for each cell separately, for ligand concentration of [L] = 5,000 and for all combinations of binding affinity (*K*_*A*_ = 100, 10, 1) and diffusion coefficients (*D* = 2.5 × 10^−6^, 2.5 × 10^−5^, 2.5 × 10^−4^ mm^2^/s). For each case (n = 3) three simulations were performed, and the average values are shown. All s.d. values were smaller than 5% and were not shown for clarity of images.

We analyzed differences in cumulative receptor saturation (% of *B*_*fast*_ – % of *B*_*slow*_) for each cell separately through the entire space of diffusion coefficient and affinity values. The relative differences in recorded receptor saturation reached up to 10% and, in the majority of cases, were in favor of the fast release scheme. This is indicated in Fig. 3a–d by the color-coded circles that differ at most by one scale color between the charts that correspond to fast and slow release schemes, respectively. A representative example with more detailed analysis is shown in Fig. 6. Among all considered cases ([*L*] = 5,000, *D* = 2.5 × 10^−6^, 2.5 × 10^−5^ and 2.5 × 10^−4^ mm^2^/s; and *K*_*A*_ = 1, 10 and 100), the highest disparities of saturation were observed for ligand molecules of low affinity. In these cases, ligands released from the vasculature in larger amounts over shorter periods of time (fast release) showed better ligand recognition and binding due to increased availability of the ligand molecule in the vicinity of the receptor at any given time. Similarly, the smallest differences were observed for the ligands with high binding affinity due to efficient receptor-ligand recognition. The cell receptors located near the vasculature were more saturated if the ligand was released fast with slow diffusion. However, the cell receptors located further from the vasculature became more saturated by the ligand that was released fast with fast diffusion through the interstitial space. In general, these results indicate that a fast release scheme of a ligand leads to more successful delivery to each cell and to more effective saturation of cell receptors.

## Discussion

We presented a computational approach for quantifying targeting ligands specificity in the heterogeneous tumor tissues. In particular, we investigated the penetration depth of ligand molecules through the tumor interstitial space, the process of ligand binding to cell membrane receptors, receptor saturation and ligand internalization. Starting with the biophysical and biochemical properties of pancreatic cells expressing the TLR2 receptor, and the TLR2-specific ligand, we explored the model parameter space with a goal to identify properties of both the ligand and the cell to achieve high receptor saturation. To do so, we progressively bridged multiple scales of resolution: from a tissue to a cell, to the membrane receptor sites. We considered various biochemical and biophysical properties of ligand molecules that affect their diffusion, such as molecule size and mass (spanning a range of small molecules, peptide-conjugates, antibodies and nanoparticles), or distinct hydrophilic/hydrophobic and solubility properties of these compounds. Moreover, we also accounted for diverse ligand binding affinities to the cell membrane receptors including high, moderate and low probabilities of association.

**Figure 7:**
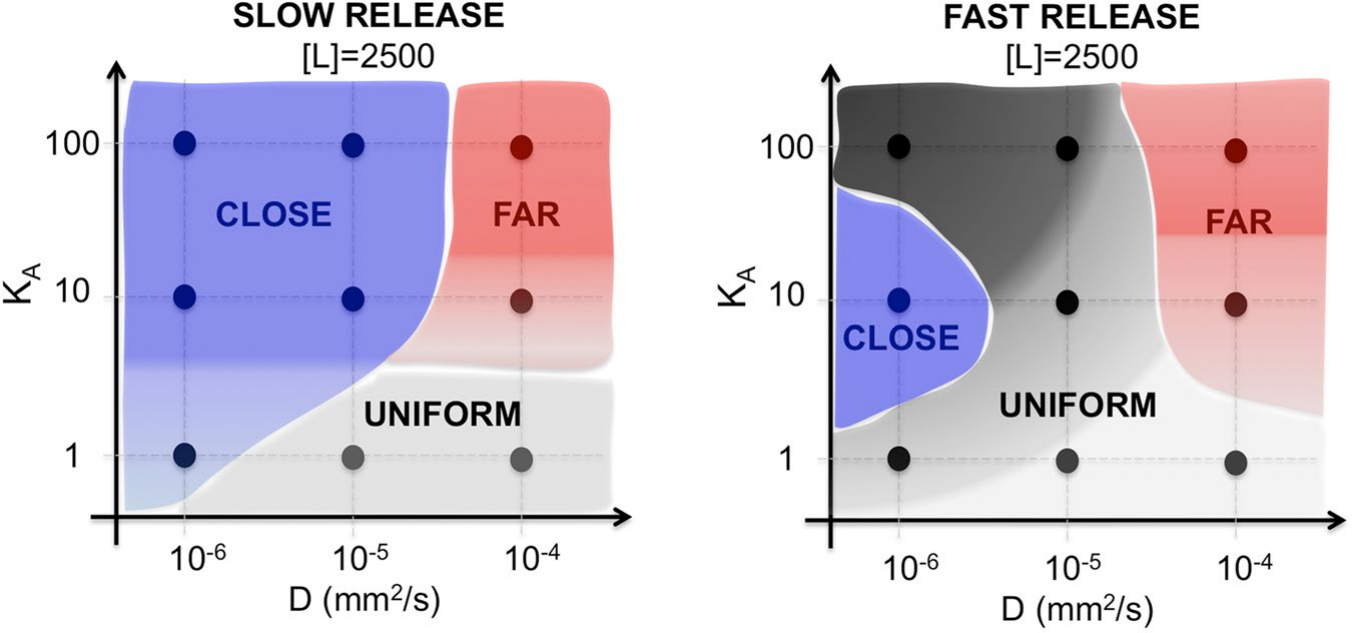
Graphical summary of key finding from *microPKPD* simualtions. At-a-glance analysis of spatial distribution of predominantly saturated cells in relation their distance from the vasculature. Nine combinations of ligand diffusivity and binding affinity for a ligand concentration of [L] = 2,500 and two release schemes are considered. Colors indicate areas in which cell receptor saturation was higher: either close to a vessel (cells #1–4, blue region), or far from a vessel (cells #5–8, red region), or similar saturation in the whole domain (grey region). Color shades indicate whether saturation was partial (light shade) or full (dark shade).

Our results showed that binding efficacy is not directly proportional to the amount of ligand entering the tissue. In fact, comprehensive examination of a broad range of properties of targeting agents, as well as their various release rates and dosages demonstrated that for fast diffusing agents the cells located near the vasculature remained undertreated and their receptors unsaturated even for higher drug concentrations. In contrast, the uptake rate by the distant cells in these cases was considerable higher and their receptors reached the elevated saturation levels much faster than cells in close proximity to the ligand source. On the contrary, the low mobility ligands showed better receptor saturation for cells near the vessel, as well as better penetration of tight spaces between the cells. Thus, the therapeutic agents may need to be designed differently depending on location of tumor cells they aim to target. For example, to treat the fast-growing cells located near the vasculature the slowly diffusing agents may be beneficial. In contrast, for the dormant cells in poorly vascularized region, the highly mobile agents may be preferential. A graphical “at-a-glance” analysis showing in which tissue regions cells will be predominantly saturated for a given combination of ligand properties is presented in Fig. 7.

Our analysis of two distinct release schemes indicated that ligands with moderate binding affinity to cell membrane receptors but that are rapidly released from the vasculature could reach similar saturation efficacy as high affinity ligands extravasating at slower rate. In-depth examination of fast and slow release schemes that took into account various combinations of ligand diffusivity and binding affinity indicated that a fast release scheme leads to more successful delivery of ligands within a tissue with heterogeneous receptor expression. It also results in a more effective saturation of cell receptors. Therefore, modification of biochemical and biophysical properties of the ligand, such as changing its mass or solubility, may increase the rates with which particles are released from vasculature. Additionally, the mode of delivery may be altered to achieve faster receptor saturation. For example, a high dose/one-time injection may lead to quicker saturation of receptors and enhanced drug uptake. In some cancers local injection directly to the tumor site may be beneficial. The latter procedures are being tested computationally^40,41^ and in ongoing clinical trials^42–45^.

In conclusion, for targeted therapies, it is critical to be able to assess target engagement of the drug *in vivo*, since *in vitro* experiments may not faithfully represent such a process. The integrative approach proposed here combines *in vivo* experiments, intravital imaging and mathematical modeling as a step in that direction. The presented computational protocols, although simplified to three orders of affinity and diffusion values, and two release schemes, can straightforwardly be adjusted to various cases of ligand-receptor interactions, and specific cancer tissue architectures. Such a simulation-based tuning of the *a priori* known physicochemical properties of targeting ligands and their release schedules might help reduce discrepancies in the therapeutic agent uptake by individual cells within particular extracellular matrix environments, tumor topologies and receptor expression levels *in vivo*. Furthermore, since the spatial organization of tumor components can be obtained from patient biopsy samples, our *microPKPD* model may provide a way to design personalized treatment protocols^32,46^.

## Electronic supplementary material

Supplementary Material
