# Supplementary material for: Targeting Ligand Specificity Linked to Tumor Tissue Topological Heterogeneity via Single-Cell Micro-Pharmacological Modeling
Source: Sci Rep. 2018 Feb 26;8:3638. doi: 10.1038/s41598-018-21883-z (PMC5827036; doi:10.1038/s41598-018-21883-z)
Supplement: Supplementary file 1 — Supplementary Material [file 41598_2018_21883_MOESM1_ESM.pdf]

Targeting Ligand Specificity Linked to Tumor Tissue Topological Heterogeneity via Single-Cell Micro-Pharmacological Modeling

Aleksandra Karolak, Veronica C. Estrella, Amanda S. Huynh, Tingan Chen, Josef Vagner, David L. Morse, Katarzyna A. Rejniak

--- Supplementary Material ---

Supplementary Material S1. Computational model parameters

Supplementary Table S1. Computational and physico-chemical parameters used in the *microPK/PD* model.

Parameter	Symbol	Value	Unit
Time step	Δt	0.1	s
Proximity distance	r_{min}	250	nm
Single active site saturation level	B	5	NA
Initial tissue saturation	B_0	0	NA
Maximum tissue saturation	B_{max}	1430	NA
Affinity constant	K_A	1, 10, 100	%
Virtual dissociation constant (inverse of K_A)	K_D	100, 10, 1	%
Diffusion coefficient	D	$2.5x10^{-4}$, $2.5x10^{-5}$, $2.5x10^{-6}$	mm^2/s
Temperature	T	300	K
Boltzmann constant	k_B	$1.38x10^{-23}$	$kg.m^2/s^2/K$
Viscosity	η	$1.89x10^{-3}$	kg/m.s
Molecule radius	R	5	nm

Supplementary Material S2. Estimating the number of cell membrane receptors.

In vitro receptor number determination.

The number of TLR2 receptor molecules expressed on the surface of the SU.86.86 pancreatic adenocarcinoma cells was calculated using an adapted version of the binding assay, as previously described[1,2]. Increasing amounts of Eu-DTPA-TLR2L were added to cells in 96-well plates. To test nonspecific binding, cells were pre-incubated with 1 μM TLR2 agonist Pam2CSK4 (InvivoGen) prior to the addition of labeled ligand. The data were fit with GraphPad Prism software using the nonlinear regression, one-site binding equation. Each data point indicates the average of four assays with 4 replicates, with error bars indicating the standard error of the mean. GraphPad Prism was used to plot the standard curves. The standard curves were then used to determine the amount of ligand present at the B_{max} obtained in the saturation binding assay. The average number of cells per well at the end of the assay was calculated. To determine the receptor number, the following equation was used: (Europium amount for B_{max} (mole) / avg cell number per well) x 6.023 x 10^{23}= receptor number per cell.

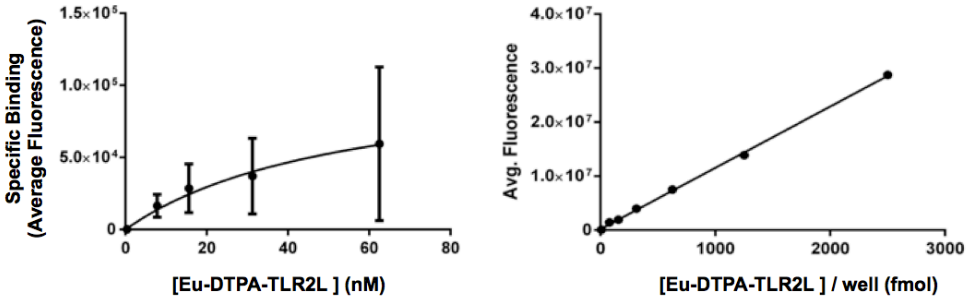

Supplementary Figure S2. In vitro receptor number determination. A) The saturation binding curve for the SU.86.86 cells determined a K_D of 55 ± 27 nM and B_{max} of 110,534 + 31,081 AFU (R^2 =0.9663, n=5 assays x 4 replicates). B) The linear regression plot of fluorescence versus ligand concentration for Eu-DTPA-TLR2L (y=102862X + 4.2741), in which the B_{max} value corresponds to 1.0745 fmol per well for SU.86.86 cells. The endogenous TLR2 expressing human pancreatic adenocarcinoma cell line, SU.86.86 has 19,300 $\pm$ 5428 TLR2 molecules expressed on the cell surface.

Estimation of the maximal number of receptor sited on the cell circumference in silico

Given that the maximum number of TLR2 receptors per cell of SU.86.86 line (each cell being on average 20μm in diameter) was determined to be 19,300 ± 5428 (Supplementary Figure S2), we estimated that there are up to a maximum of 250 receptors present on the cell circumference. For the purpose of mathematical modeling, we consider that an active virtual site corresponds to a cluster of five biological receptors, and thus each such a site can accept up to five pseudo-ligand molecules. The number of active virtual sites (defining cell boundary) on the membrane of each cell and the number of corresponding receptors (pseudo-receptors) are presented in the Table S2.

Supplementary Table S2: Number of receptors per each cell in a virtual tumor tissue.

Cells	1	2	3	4	5	6	7	8
Virtual active sites per cell (A_s)	32	40	31	30	43	43	33	35
Total number of pseudo-receptors per cell (A_s * 5)	160	190	155	150	215	215	165	175
Total number of pseudo-receptors per tissue								1430

Supplementary Material S3. Experimental determination of Cy5-TLR2L binding affinity for TLR2.

Cell Culture

SU.86.86 human pancreatic adenocarcinoma cells (ATCC CRL-1837) were grown in RPMI 1640 media (Life Technologies Gibco) supplemented with 10% FBS (VWR Seradigm Radnor, Pennsylvania) at 37°C and 5% CO2. Endogenous expression of TLR2 in SU.86.86 cells was characterized previously[2] and, for the study herein, expression was confirmed in using time-resolved fluorescence saturation binding assays (below). Prior to and upon completion of experiments, cell lines were authenticated using short tandem repeat (STR) DNA typing according to ATCC's guidelines[3].

In Cyto Europium Time-Resolved Fluorescence (TRF) Competition Binding Assays

As previously described, europium TRF competition binding assays were performed to test the TLR2 binding specificity of the Cy5-TLR2L ligand using the SU.86.86 cell line [2]. Cells were grown in 96-well plates for 2 days, reaching approximately 80% confluency. On the day of the experiment, the cell culture medium was aspirated and 50 µL of nonlabeled test ligand was added in a series of decreasing concentrations (1 µM to 0.01 nM) followed by 50 µL of the competing europium-labeled TLR2 ligand (Eu-DTPA-TLR2L), Eu-DTPA-Mpr-PEGO-Cys(*S*-[2,3-bis(palmityol)oxy-(R)-propyl])-Gly-DSer-PEGO-NH_2, at a fixed concentration of 90 nM. Cells were incubated with labeled and unlabeled ligands for 1 h at 37 °C. Following incubation, cells were washed three times to remove unbound ligand. Next, 100 µL of DELFIA enhancement solution (PerkinElmer) was added to each well. Cells were incubated for 30 min at 37 °C prior to reading. The plates were read on a PerkinElmer VICTOR X4 multilabel reader using the standard europium TRF protocol. To determine the mean K_i, statistical analysis was performed using the one-site competition fit using least squares in GraphPad Prism software.

Experimental Cy5-TLR2L binding affinity

Lanthanide-based TRF competition binding assays were performed using SU.86.86 human pancreatic cancer cells that endogenously express TLR2. The binding affinity of Cy5-TLR2L for TLR2 was determined to be 61 nM K_i (n = 3 assays, R^2 = 0.88). See Supplementary Figure S3 below. The binding affinity for the Cy5 conjugate version is similar to our published 67 nM K_i affinity of our IRDye800CW conjugate for TLR2[2].

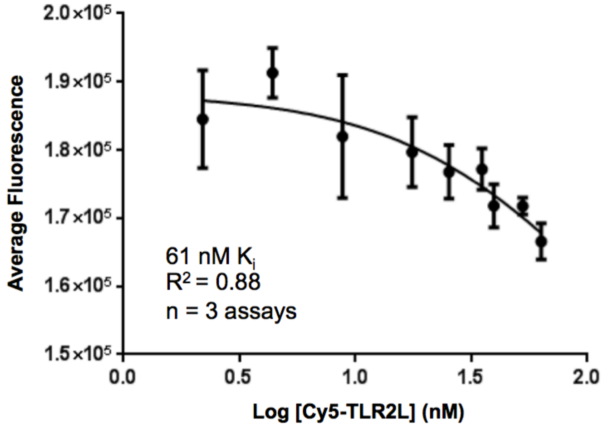

Supplementary Figure S3. One-site competition line-fit of data generated by the in cyto TRF competition binding assays. Competition binding assays were performed in which increasing concentrations of Cy5-TLR2L were added in the presence of 90 nM Eu-DTPA-TLR2L using SU.86.86 cells.

<u>Supplementary Material S4. Estimating value of the effective diffusion coefficient for Cy5-TLR2L molecules.</u>

The effective diffusion coefficient *D* for the molecules of Cy5-TLR2L complex was calculated using the Stokes-Einstein formula given by Equation S4.1:

$$D = \frac{k_B T}{6\pi R \eta}, \qquad \text{(S4.1)}$$

where k_B is Boltzmann constant, η is solvent viscosity, R is the radius of diffusing particle and T is temperature (see Table S1 for parameter values).

Thus,

$$[D]=\left[\frac{\frac{kg\times m^2}{s^2\times K}\times K}{m\times\frac{kg}{m\times s}}\right]=\left[\frac{m^2}{s}\right]=\left[10^6\times\frac{mm^2}{s}\right],$$

$$D=\frac{1.38\times10^{-23}\times300}{6\times3.14\times5\times10^{-9}\times0.00189}\left[\frac{m^2}{s}\right]=2.5\times10^{-11}\left[10^6\times\frac{mm^2}{s}\right]=2.5\times10^{-5}\left[\frac{mm^2}{s}\right]. \qquad (S4.2)$$

Since *D* defines the flux of particles through the space in time, in 2D environment *D* has units of area/time (Equation S4.2).

Supplementary Material S5. Cy5-TLR2L Synthesis, Purification and Analysis.

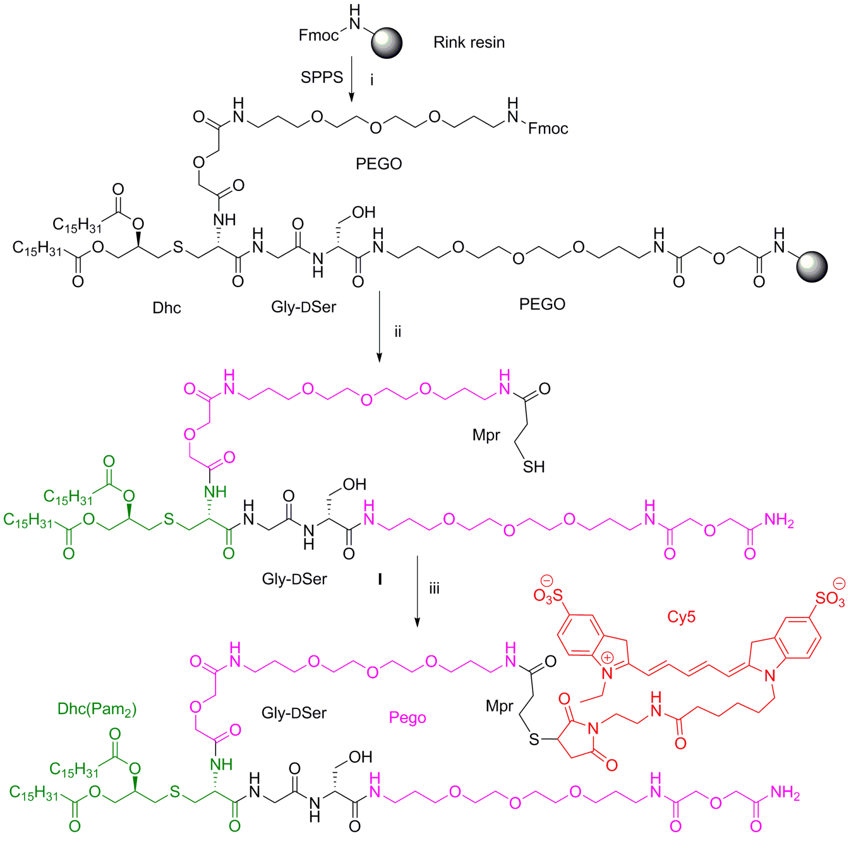

Cy5-Mpr-Pego-Dhp(Pam2)-Gly-DSer-Pego

Supplementary Scheme S5. Synthetic Route for Cy5-Mpr-PEGO-Cys(S-[2,3-bis(palmityol)oxy-(R)-propyl])-Gly-DSer-PEGO-NH_2 (Cy5-TLR2L). Precursor was prepared on solid support as previously described[2]. Then, [i] Fmoc/tBu synthesis continued as follows: a) Piperidine/DMF (1:4) for Fmoc deprotection; b) Fmoc-aa-OH (3eq), HOBt (3eq), DIEA (6eq), and HBTU (3eq) in DMF for amino acid or Fmoc-Pego couplings [ii] TFA-scavengers cocktail (90% trifluoroacetic acid, 5% water, 5% triisopropylsilane) for 2 hrs; [iii] Cy5 maleimide (1.05 eq) in DMF/PBS buffer for 12hrs.

Cy5-TLR2L is similar to the previously published compound IRDye800CW-Mpr-PEGO-Cys(*S*-[2,3-bis(palmityol)oxy-(R)-propyl])-Gly-DSer-PEGO-NH_2, except that the far-red Cy5 dye (Lumiprobe, Hallandale Beach, FL) was conjugated instead of the near-infrared dye IRDye800CW[2] to generate Cy5-Mpr-PEGO-Cys(*S*-[2,3-bis(palmityol)oxy-(R)-propyl])-Gly-DSer-PEGO-NH_2 (Cy5-TLR2L), see Scheme S7. Compounds were fully deprotected and cleaved from the resin by treatment with 91% TFA (3% water, 3% EDT, and 3% TA) or 90% TFA (5% water, 5% TIS). After ether extraction of scavengers, compounds were purified by HLPC and/or size-exclusion chromatography (Sephadex G-25, 0.1 M acetic acid) to >95% purity. Cy5-TLR2L (2318.2 MW) was analyzed for purity by analytical HPLC and MS by ESI or MALDI-TOF.

Supplementary Material S6. Animals.

All procedures were in compliance with the Guide for the Care and Use of Laboratory Animal Resources (1996), National Research Council, and approved by the Institutional Animal Care and Use Committee, University of South Florida, under an approved protocol. Immunocompromised NOD/SCID mice, 22-25 g, were purchased from Charles River Inc. and housed in a clean facility with special conditions that include HEPA filtered ventilated cage systems, autoclaved bedding, autoclaved housing, autoclaved water, irradiated food, and special cage changing procedures. Mice were handled using aseptic methods including the wearing of gloves, gowns, and shoe coverings. Mice were anesthetized by inhaled isoflurane gas (flow 2-2.5 L/min) and remained anesthetized for the minimum amount of time required for imaging studies, ranging from 20 to 120 min at a time.

Supplementary Material S7. Statistical analysis of experimental and computational association kinetics

To determine virtual concentrations of the ligand that matched experimental data, a range of virtual ligand concentrations between 500 and 10,000 were applied in simulations, and the obtained data points were fitted to the association kinetics curves (Equation 2). The results for sever different concentrations of a high affinity ligand (K_A=100), with baseline diffusion coefficient (D=$2.5x10^{-5}mm^2/s$), released slowly from the vasculature are shown in Figure S7, and the full statistical analysis is presented in Table S7. Ligand pseudo-concentrations of 5,000 and 7,500 molecules produced curves that were the closest to the experimentally administered dose.

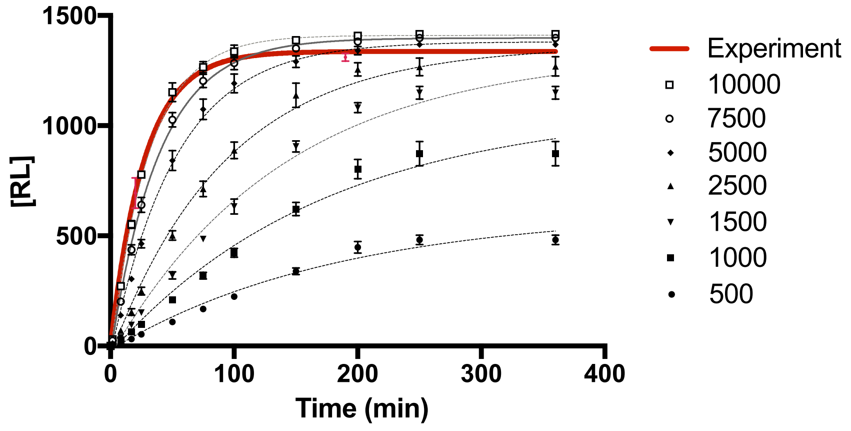

Supplementary Figure S7. In silico and in vivo determination of association kinetics curves for high affinity ligand (K_A=100), with baseline diffusion coefficient (D=2.5x10^{-5} mm^2/s) slowly released from vasculature. Each ligand concentration is indicated by a different. The experimental association kinetics curve is shown in red. For each case considered, (n=3) simulations were repeated, and the averaged value is shown. The error bars indicate s.d. Curve fitting was performed with GraphPad Prism software using the one-phase association kinetic method[4].

Supplementary Table S7. Statistical comparison between association kinetics for experimental data and computational simulations for ligand concentrations of [L]=5,000 and [L]=7,500 from GraphPad Prism software.

**Experiment to simulation**	**[L]=5,000**	**[L]=7,500**
**Best-fit values**		
B_0	-15.49	-19.43
B_{max}	1360	1368
K	0.02074	0.02835
T	48.21	35.27
Half-time	33.41	24.45
Span	1376	1387
**Std. Error**		
B_0	23.49	19.52
B_{max}	20.12	14.16
K	0.001287	0.001337
Span	28.34	22.84
**95% CI (asymptotic)**		
B_0	-62.8 to 31.82	-59.14 to 20.28
B_{max}	1320 to 1401	1339 to 1397
K	0.01815 to 0.02334	0.02563 to 0.03107
T	42.85 to 55.09	32.18 to 39.02
Half-time	29.7 to 38.18	22.31 to 27.04
Span	1319 to 1433	1341 to 1434
**Goodness of Fit**		
R^2	0.9813	0.9913
Absolute Sum of Squares	264355	93703
Sy.x	76.65	53.29

Supplementary Material S8. Statistical analysis of computational binding kinetics

To ascertain whether the simulated data fit the classical binding kinetics equations without imposing the values for their parameters, the outputs from association kinetics equations were determined for various ligand concentrations before their plateaus were reached following the method described in [5]. The fitting results to Equation 3 and Equation 4 are presented in Figure S8 and the full statistical analysis is presented in Table S8.

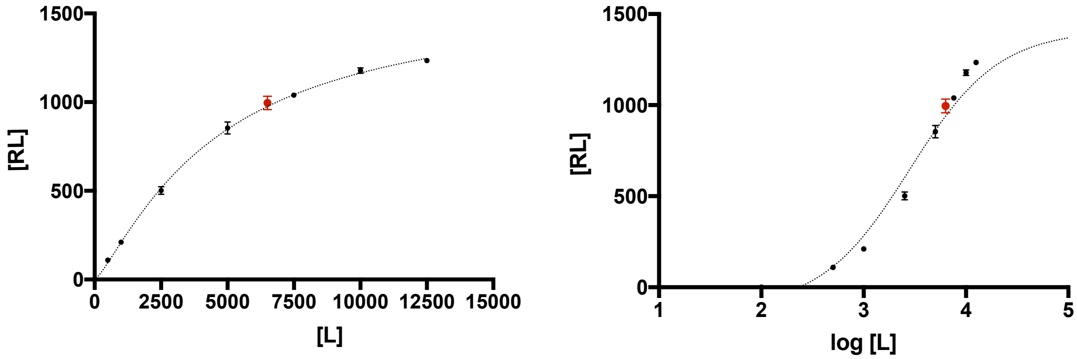

Supplementary Figure S8. In silico determination of binding kinetics curves for high affinity ligand (K_A=100), with baseline diffusion coefficient (D=2.5x10^{-5} mm^2/s) slowly released from vasculature. (left) Fitting to Equation 3; (right) Fitting to Equation 4. The 90% saturation values from experimental and computational data were determined from Figure S7 (experimental value is indicated in red). Each measurement was repeated 3 times (n=3) and the average values are shown. The error bars indicate s.d. Curve fitting was performed with GraphPad Prism software[4].

Supplementary Table S8. Statistical analysis from GraphPad Prism software for fitting simulated data to the binding kinetics Equation 3 (left) and Equation 4 (right).

One site - Specific binding		One site - Fit logIC50	
**Best-fit values**		**Best-fit values**	
B_{max}	1599	B_0	-116.7
h	1.248	B_{max}	1411
K_D	4543	LogIC50	3.451
		IC50	2823
**Std. Error**		**Std. Error**	
B_{max}	48.35	B_0	46.83
h	0.04437	B_{max}	47.04
K_D	271.6	LogIC50	0.06372
**95% CI (asymptotic)**		**95% CI (asymptotic)**	
B_{max}	1499 to 1700	B_0	-213.3 to -19.99
h	1.155 to 1.34	B_{max}	1314 to 1508
K_D	3978 to 5108	LogIC50	3.319 to 3.582
		IC50	2085 to 3821
**Goodness of Fit**		**Goodness of Fit**	
R^2	0.9986	R^2	0.9702
Absolute Sum of Squares	7184	Absolute Sum of Squares	185473
Sy.x	18.5	Sy.x	87.91

Supplementary Material S9. Association kinetics for fast extravasating ligand molecules.

Correspondingly to the estimation of association kinetics for the slow release scheme (Figure 2), we performed the fitting for all simulations in which the ligand was released fast (Figure S9). As expected, the association curves for all considered concentrations are characterized by high steepness before their plateaus are reached. Similarly to slow release kinetics, for all rapidly released ligands possessing high affinity (K_A=100) saturation levels are not strongly dependent on the diffusion coefficient *D*. Nonetheless, the progression of saturation slows down with decreased diffusion coefficient values. For moderate affinity (K_A=10) the final levels of saturation decrease with increasing *D*, similarly to Figure 2. For low value of binding affinity (K_A=1), the correlation between affinity and diffusivity plays a stronger role, and the maximum saturation levels of receptor are not reached for any of the concentrations considered as it was noticed in simulations with ligand slow release. The full considered parameter space includes three values of the diffusion coefficient *D*: 2.5×10^{-6}, 2.5×10^{-5}, and 2.5×10^{-4} mm^2/s; three values of binding affinity K_A: 100, 10, and 1; and ligand concentrations values [*L*] between 500 and 10000. All simulated results are shown in Figure S9.

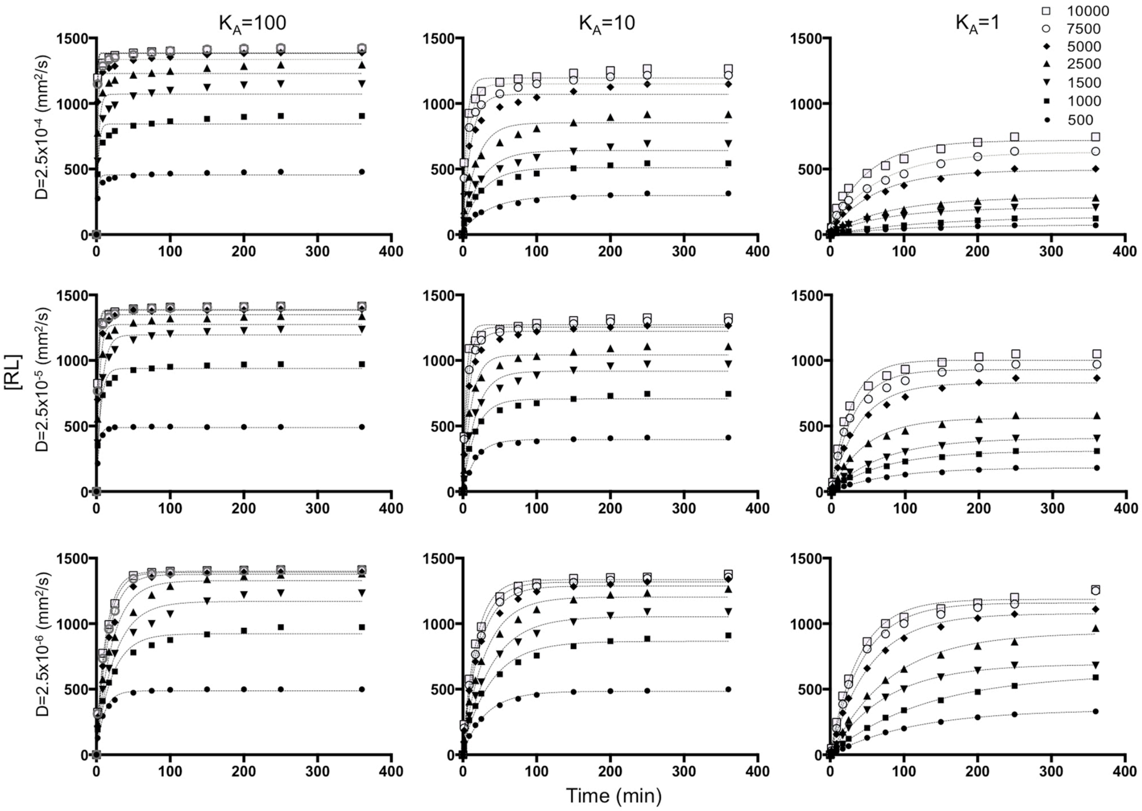

Supplementary Figure S9. Association kinetics for fast extravasating ligand molecules. For each case considered (n=3) simulations were performed and the averaged value in shown. All s.d. values are smaller than 5% and are not presented for better visual clarity.

Supplementary Material S10. Comparison of logK$_D$ between three affinity values.

Dissociation constant (K_D) is a universally used equilibrium constant that in binding kinetics identifies the concentration of ligand for which 50% of receptors are saturated by this ligand and 50% of ligand molecules are unbound. K_D is an inverse of K_A and any of these two terms allows for identification of ligand concentration for which 50% of the molecules dissociated or associated with receptors, respectively. Values of both constants are commonly presented in logarithmic scale. Table S10 summarizes numerical outcomes of the fitting to binding kinetics curves from simulations with D=2.5x10^{-5} mm^2/s for all affinity values within both release schemes. In agreement with our previous discussion on the binding kinetics for fast and slow release schemes illustrated in Figure 3, the K_D values for fast release moderate affinity and slow release high affinity confirm observed similarity.

Supplementary Table S10. Comparison of logK$_D$ between three affinity values and D=2.5x10^{-5} mm^2/s.

	Fast release			Slow release		
K_A	100	10	1	100	10	1
LogK$_D$	2.99	3.57	3.79	3.55	3.64	3.84
S.D.	0.03	0.07	0.14	0.10	0.12	0.15

References.

1 Handl, H. L., Vagner, J., Yamamura, H. I., Hruby, V. J. & Gillies, R. J. Development of a lanthanide-based assay for detection of receptor-ligand interactions at the delta-opioid receptor. *Anal Biochem* **343**, 299-307, doi:10.1016/j.ab.2005.05.040 (2005).

2 Huynh, A. S. *et al.* Novel Toll-like Receptor 2 Ligands for Targeted Pancreatic Cancer Imaging and Immunotherapy. *J Med Chem* **55**, 9751-9762, doi:10.1021/jm301002f (2012).

3 Reid, Y., Storts, D., Riss, T. & Minor, L. in *Assay Guidance Manual* (eds G. S. Sittampalam *et al.*) (2004).

4 GraphPadPrism. (https://www.graphpad.com/scientific-software/prism/, 2016).

5 Hulme, E. C. & Trevethick, M. A. Ligand binding assays at equilibrium: validation and interpretation. *Brit J Pharmacol* **161**, 1219-1237, doi:10.1111/j.1476-5381.2009.00604.x (2010).
